# Higher hospital volume is associated with lower mortality for patients with cardiogenic shock and mechanical circulatory support

**DOI:** 10.1002/ejhf.70025

**Published:** 2025-08-31

**Authors:** Angela Dettling, Caroline Kellner, Jonas Sundermeyer, Benedikt N. Beer, Lisa Besch, Marvin Kriz, Stefan Kluge, Paulus Kirchhof, Stefan Blankenberg, Benedikt Schrage

**Affiliations:** ^1^ Department of Cardiology University Heart and Vascular Center Hamburg, University Medical Center Hamburg‐Eppendorf Hamburg Germany; ^2^ German Center for Cardiovascular Research (DZHK) partner site Hamburg/Kiel/Lübeck Hamburg Germany; ^3^ Center for Population Health Innovation (POINT) University Heart and Vascular Center Hamburg, University Medical Center Hamburg‐Eppendorf Hamburg Germany; ^4^ Department of Clinical Science and Education, Södersjukhuset Karolinska Institutet Stockholm Sweden; ^5^ Department of Intensive Care Medicine University Medical Centre Hamburg‐Eppendorf Hamburg Germany

**Keywords:** Cardiogenic shock, Mechanical circulatory support, Mortality, Epidemiology, Case volume, Cardiogenic shock networks

## Abstract

**Aims:**

Mortality for cardiogenic shock (CS) remains high. To improve outcomes, centralization of treatment in specialized centres, especially those with expertise in mechanical circulatory support (MCS), has been recommended. High‐volume centres may be able to provide standardized, better care. We analysed associations between centre volume and outcomes in Germany, a large country with multiple types of CS centres.

**Methods and results:**

Based on data from all CS patients treated in Germany from 2017–2021, the association between annual CS/MCS hospital volume and in‐hospital mortality was assessed using adjusted Cox‐regression, and spline plots were used to assess case thresholds. Overall, 220 223 CS patients underwent treatment at 1232 hospitals; 435/1232 (35%) of these performed MCS therapy, although only few hospitals (60/435, 14%) performed >25 MCS cases per year on average. Treatment at hospitals with a higher annual volume of CS and MCS cases was associated with a significantly lower mortality risk as compared to hospitals with a lower volume (upper third vs. lower two‐thirds; CS: hazard ratio [HR] 0.92, 95% confidence interval [CI] 0.91–0.94; *p* < 0.001; MCS: HR 0.80, 95% CI 0.76–0.84; *p* < 0.001). These associations were continuous without a detectable ceiling effect, with spline plots suggesting case thresholds of at least 90 CS cases/25 MCS cases per year.

**Conclusions:**

Care for patients with CS treated with and without MCS is associated with lower in‐hospital mortality in hospitals that manage high volumes of CS and MCS. This analysis indicates that centralization of CS care in specialized centres treating high volumes of patients with CS and MCS might improve outcomes.

## Introduction

Cardiogenic shock (CS) is a life‐threatening condition with an average annual hospitalization of 44 044 patients in Germany and a mortality rate exceeding 50% at 30 days.^1^, ^2^ Rapid restoration of adequate circulation is a key component of successful therapy. Current evidence and treatment guidelines recommend to consider the use of mechanical circulatory support (MCS) devices in highly selected cases to maintain life‐prolonging circulation as a bridge to recovery and even to alleviate cardiac damage.^3^, ^4^, ^5^, ^6^, ^7^ However, the application of MCS systems as well as the successful identification and therapy of underlying causes leading to CS are highly complex and require a well‐trained and well‐organized interdisciplinary team.^8^ CS itself often leads to multi‐organ failure and use of MCS systems is associated with a high risk of periprocedural complications, both complicating clinical practice.^9^ As a result, the application of MCS is highly resource demanding and associated with high healthcare costs.^10^, ^11^, ^12^

Evidence suggests that hospital volume is a critical factor influencing patient outcomes for several cardiovascular procedures.^13^, ^14^ Higher volume centres possess specialized resources and experienced multidisciplinary teams capable of implementing complex, protocol‐driven care that may improve survival rates and reduce complications.^15^ Nevertheless, decentralized cardiovascular care in multiple hospitals remains common. Robust nationwide individual‐level data on CS treatment and use of MCS are needed to gather information on the impact of centre volume on outcomes in CS. A good model country to study this is Germany, a large country with near‐universal healthcare coverage but regionally diverse patterns of hospital care, resulting in a landscape with multiple types of CS centres.

To determine the effect of hospital volume on outcomes in CS patients treated with and without MCS, we therefore analysed a unique, complete and contemporary database of all consecutive patients with CS treated with and without MCS in Germany. The analysis addresses the following questions: (1) what is the current landscape of CS treatment and MCS use in Germany; (2) is there an association between CS and MCS hospital volume and in‐hospital mortality; and (3) can optimal case thresholds for CS treatment and MCS use be estimated.

## Methods

### Data source

The Research Data Centre of the Federal Statistical Office and Statistical Offices of the Federal States in Germany (http://www.destatis.de) routinely collects comprehensive data of all patients treated at German hospitals. This database includes records of all diagnoses, medical procedures, demographic data, hospital data and in‐hospital mortality. Thorough data collection is ensured, due to its linkage to reimbursement processes, which mandates submission and auditing. Diagnoses are coded using the German modification of the International Statistical Classification of Diseases and Related Health Problems, 10th Revision (ICD‐10‐GM), while procedures are cataloged using the German Operational and Procedural Codes (OPS). These patient‐level data are fully anonymized and centrally stored at the Research Data Centre of the Federal Statistical Office and Statistical Offices of the Federal States in Wiesbaden, Germany. The investigators accessed only anonymized statistical reports, without direct access to individual patient‐level data. This procedure is covered by a general ethics approval applied for by the Federal Statistical Office and Statistical Offices of the Federal States in Germany, which waives individual ethics committee application as well as a need for an individual informed consent, and was performed in accordance to the Declaration of Helsinki.

### Population

Between 2017 and 2021, all patients treated at a German hospital with the diagnosis of CS (ICD‐10‐GM code R57.0; either present at hospital admission or developed during the hospital stay) were included. Patients under the age of 18 were excluded from the analysis. For those included, data on coexisting diseases and treatments were obtained via ICM‐10‐GM codes or OPS codes, as specified in online supplementary *Table Appendix* *S1*.

### Statistical analysis

The Research Data Centre of the Federal Statistical Office and Statistical Offices of the Federal States handled the data export on our behalf. The authors developed a detailed analysis plan and developed a dedicated R code (R statistical software, version 4.0.3) based on dummy data. This code was then run by the team of the Research Data Centre of the Federal Statistical Office and Statistical Offices of the Federal States on the local database, and aggregated results were sent back to the authors, who verified for consistency and prepared for publication. Individual patient‐level data always remained at the Research Data Centre of the Federal Statistical Office and Statistical Offices of the Federal States and were never available to the authors.

Categorical variables are shown as frequency and percentage and compared using the Chi‐squared test, continuous variables are shown as mean and standard deviation and compared using one‐way analysis of variance. The annual case volume of CS and the use of MCS devices per hospital, including veno‐arterial extracorporeal membrane oxygenation (VA‐ECMO), micro‐axial flow pumps (mAFP) and intra‐aortic balloon pump (IABP), were calculated based on cases reported from January 1 to December 31 of each year (if a patient was treated with multiple devices, a hierarchical order was used, with VA‐ECMO at the highest and IABP at the lowest rank). For both variables, hospitals were categorized based on average annual case volume over the period from 2017 to 2021 into high volume centres (3rd tertile of CS cases or MCS use) versus intermediate‐low volume centres (1st and 2nd tertile of CS cases or MCS use). Patient‐level data were stratified by this division of annual case volume of CS and MCS use, respectively.

The primary outcome was in‐hospital mortality (time to event analysis). To evaluate the association between case volume of CS and mortality risk, as well as case volume of MCS and mortality risk, Cox proportional hazard regression models were fitted. All regression models were adjusted for age, sex, acute myocardial infarction, cardiopulmonary resuscitation in all analyses, and additionally for MCS use in the analysis on CS volume. Lastly, to evaluate minimal case thresholds for CS and MCS, natural cubic spline plots with 4 degrees of freedom were built. All spline plot analyses were adjusted as described above. Hazard ratios (HR) and 95% confidence intervals (CI) are reported, with a p‐value of <0.05 deemed statistically significant.

## Results

### Patient characteristics

Between 2017 and 2021, a total of 220 223 patients with CS were treated in Germany. The mean age of the study population was 71.3 years (± 13.6), with 80 298 (36.5%) patients being female. Acute myocardial infarction was the most common cause of CS, affecting 92 186 (41.9%) patients, and 110 551 patients (50.2%) presented with cardiac arrest. MCS devices were used in 27 836 (12.6%) patients. The most frequently used MCS device was VA‐ECMO, in 14 390 (6.5%) patients, followed by mAFP in 12 757 (5.8%) patients, and IABP in 4607 (2.1%) patients. Additional baseline characteristics of the overall cohort are detailed in *Table* *1*.

**Table 1:** Patient baseline characteristics for the overall cohort and stratified by the 5‐year mean annual cardiogenic shock volume of the treating hospital (intermediate‐low vs. high)

	All patients (*n* = 220 223)	Patients treated at intermediate‐low volume CS hospitals (<33.2 cases on average) (*n* = 38 656)	Patients treated at high volume CS hospitals (≥33.2 cases on average) (*n* = 181 567)
Demographics			
Age, years	71.3 (13.6)	74.0 (12.3)	70.8 (13.8)
Female sex	80 298 (36.5)	15 901 (41.1)	64 397 (35.5)
Comorbidities			
Atrial fibrillation	87 673 (39.8)	15 739 (40.7)	71 934 (39.6)
Diabetes mellitus	66 080 (30.0)	12 134 (31.4)	53 935 (29.7)
Arterial hypertension	87 267 (39.6)	15 718 (40.7)	71 549 (39.4)
Dyslipidaemia	54 104 (24.6)	7655 (19.8)	46 449 (25.6)
Chronic heart failure	141 538 (64.3)	22 808 (59.0)	118 730 (65.4)
Coronary artery disease	132 544 (60.2)	19 389 (50,2)	113 155 (62.3)
History of CABG	13 965 (6.3)	2231 (5.8)	11 734 (6.5)
Peripheral artery disease	18 786 (8.5)	3058 (7.9)	15 728 (8.7)
Prior stroke	8390 (3.8)	1066 (2.8)	7324 (4.0)
Pulmonary hypertension	19 032 (8.6)	2723 (7.0)	16 309 (9.0)
COPD	24 741 (11.2)	5099 (13.2)	19 642 (10.8)
Chronic kidney disease	63 543 (28.9)	12 014 (31.1)	51 529 (28.4)
Presentation			
Acute myocardial infarction	92 186 (41.9)	14 781 (38.2)	77 405 (42.6)
Severe pulmonary embolism	9193 (4.2)	1854 (4.8)	7339 (4.0)
Acute myocarditis	945 (0.4)	87 (0.2)	858 (0.5)
Peripartum cardiomyopathy	73 (<0.1)	4 (<0.1)	69 (<0.1)
Post cardiothoracic surgery	7125 (3.2)	361 (0.9)	6764 (3.7)
Cardiac arrest	110 551 (50.2)	16 444 (42.5)	94 107 (51.8)
In‐hospital management			
Coronary angiography	96 656 (43.9)	10 542 (27.3)	86 114 (47.4)
Percutaneous coronary intervention	68 890 (31.3)	7944 (20.6)	60 946 (33.6)
CABG	10 246 (4.7)	79 (0.2)	10 167 (5.6)
Mechanical circulatory support	27 836 (12.6)	803 (2.1)	27 033 (14.9)
IABP	4607 (2.1)	198 (0.5)	4409 (2.4)
mAFP	12 757 (5.8)	491 (1.3)	12 266 (6.8)
VA‐ECMO	14 390 (6.5)	132 (0.3)	14 258 (7.9)
Renal replacement therapy	42 938 (19.5)	3619 (9.7)	39 319 (21.7)
Invasive ventilation	97 188 (44.1)	15 963 (41.3)	81 225 (44.7)

Values are shown as mean (standard deviation), or as *n* (%). Variables were compared using χ^2^ test or one‐way analysis of variance. Intermediate‐low volume of CS includes the 1st and 2nd tertile and high volume of CS includes the 3rd tertile of mean annual CS hospital volume.

CABG, coronary artery bypass graft; COPD, chronic obstructive pulmonary disease; CS, cardiogenic shock; IABP, intra‐aortic balloon pump; mAFP, micro‐axial flow pump; VA‐ECMO, veno‐arterial extracorporeal membrane oxygenation.

### Incidence and hospital case volume of cardiogenic shock and use of mechanical circulatory support

**Figure 1:**
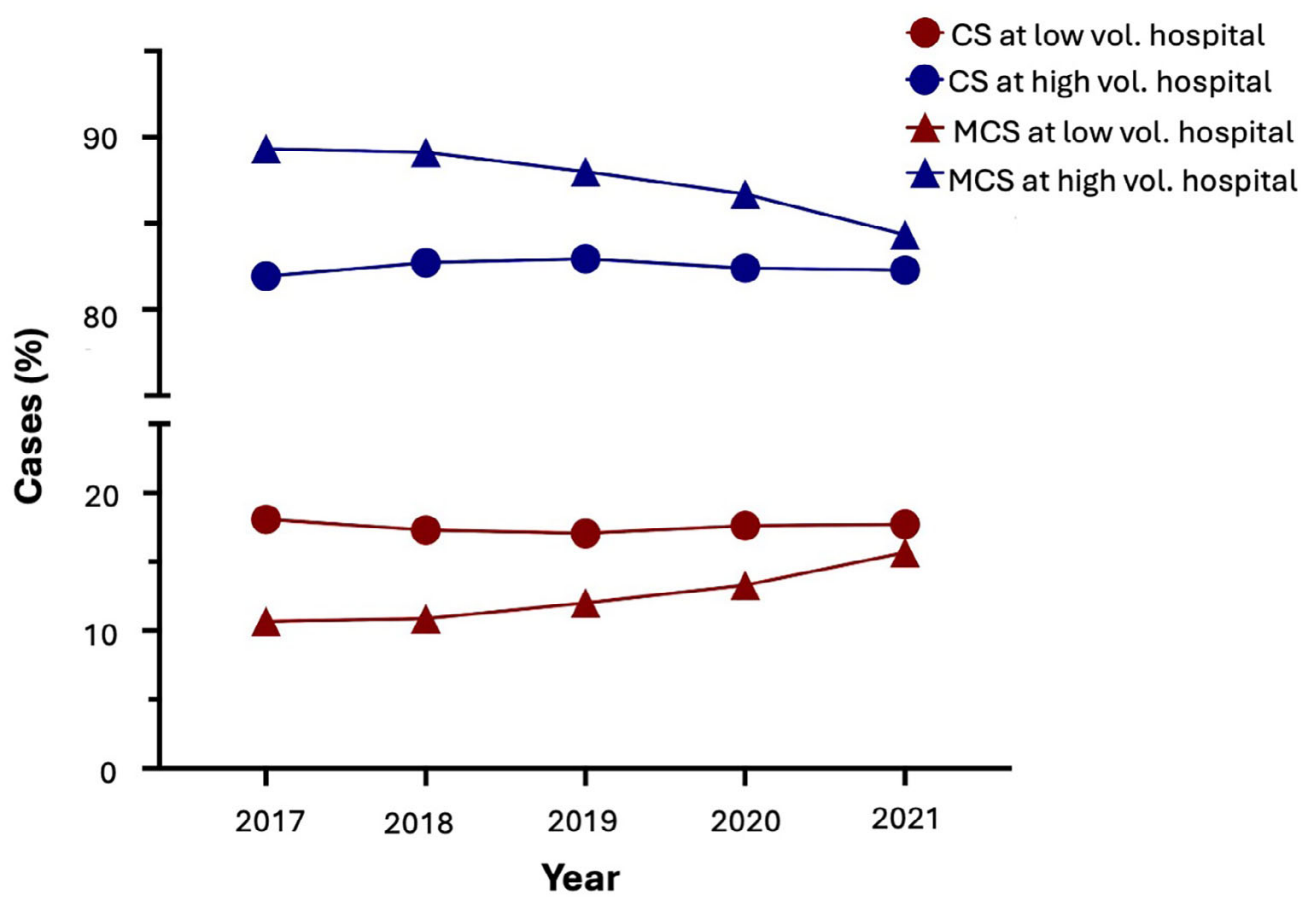
Cases treated at intermediate‐low vs. high volume hospitals for cardiogenic shock (CS) and mechanical circulatory support (MCS) over time.

Between 2017 and 2021, a total of 1232 German hospitals treated patients with CS. The overall annual volume of CS cases remained stable at approximately 44 045 cases over the years. High volume centres treated ≥33.2 CS cases per year on average, consistently accounting for more than 80% of the annual CS volume (81.9% in 2017 to 82.3% in 2021) (*Figure* *1*).

During the study period, 435 German hospitals (35.3%) used MCS for treatment of CS. The overall annual MCS use in patients with CS slightly increased from 5085 cases (11.2%) in 2017 to 5796 (13.6%) in 2021. During this period, the use of VA‐ECMO rose from 4.4% in 2017 to 5.0% in 2021, and the use of mAFP increased from 3.8% to 7.2%. Conversely, the use of IABP declined from 3.1% in 2017 to 1.4% in 2021 (online supplementary *Table* *S2*). High volume centres used MCS in ≥8.1 CS cases per year on average, accounting for more than 87.5% of the MCS case volume over the total study period, although this proportion decreased over time (89.3% in 2017 to 84.3% in 2021) (*Figure* *1*). Baseline characteristics, stratified by intermediate‐low versus high volume CS and MCS centres are presented in *Tables* *1* and *2*.

**Table 2:** Patient baseline characteristics for the overall cohort and stratified by the 5‐year mean annual mechanical circulatory support volume of the treating hospital (intermediate‐low vs. high)

	All patients (*n* = 27 836)	Patients treated at intermediate‐low volume MCS hospitals (<8.1 cases on average) (*n* = 3500)	Patients treated at high volume MCS hospitals (≥8.1 cases on average) (*n* = 24 336)
Demographics			
Age, years	64.4 (13.0)	67.0 (12.5)	64.0 (13.0)
Female sex	7280 (26.2)	912 (26.1)	6368 (26.2)
Comorbidities			
Atrial fibrillation	10 087 (36.2)	884 (25.3)	9203 (37.8)
Diabetes mellitus	7817 (28.1)	1001 (28.6)	6816 (28.0)
Arterial hypertension	10 454 (37.6)	1232 (35.2)	9222 (37.9)
Dyslipidaemia	7687 (27.6)	854 (24.4)	6833 (28.1)
Chronic heart failure	20 557 (73.9)	2229 (63.7)	18 328 (75.3)
Coronary artery disease	22 100 (79.4)	3046 (87.0)	19 054 (78.3)
History of CABG	1774 (6.4)	153 (4.4)	1621 (6.7)
Peripheral artery disease	2204 (7.9)	218 (6.23)	1986 (8.16)
Prior stroke	1801 (6.5)	91 (2.6)	1710 (7.0)
Pulmonary hypertension	3089 (11.1)	107 (3.1)	2982 (12.3)
COPD	1807 (6.5)	210 (6.0)	1597 (6.56)
Chronic kidney disease	5496 (19.7)	672 (19.2)	4824 (19.8)
Presentation			
Acute myocardial infarction	16 986 (61.0)	2675 (76.4)	14 311 (58.8)
Severe pulmonary embolism	801 (2.9)	45 (1.3)	756 (3.1)
Acute myocarditis	328 (1.2)	19 (0.5)	309 (1.3)
Peripartum cardiomyopathy	25 (0.1)	0 (0)	25 (0.1)
Post cardiothoracic surgery	3156 (11.3)	59 (1.7)	3097 (12.7)
Cardiac arrest	17 549 (63.0)	2381 (68.0)	15 168 (62.3)
In‐hospital management			
Coronary angiography	19 142 (68.8)	3215 (91.9)	15 927 (65.5)
Percutaneous coronary intervention	13 984 (50.2)	2812 (80.3)	11 172 (45.9)
CABG	5554 (20.0)	72 (2.1)	5482 (22.5)
IABP	4607 (16.6)	613 (17.5)	3994 (16.4)
mAFP	12 757 (45.8)	2499 (71.4)	10 258 (42.2)
VA‐ECMO	14 390 (51.7)	475 (13.6)	13 915 (57.2)
Renal replacement therapy	11 958 (43.0)	735 (21.0)	11 223 (46.1)
Invasive ventilation	17 112 (61.5)	2192 (62.6)	14 920 (61.3)

Values are shown as mean (standard deviation), or as *n* (%). Variables were compared using χ^2^ test or one‐way analysis of variance. Intermediate‐low volume of MCS includes the 1st and 2nd tertile and high volume of MCS includes the 3rd tertile of mean annual MCS hospital volume.

CABG, coronary artery bypass graft; COPD, chronic obstructive pulmonary disease; IABP, intra‐aortic balloon pump; mAFP, micro‐axial flow pump; MCS, mechanical circulatory support; VA‐ECMO, veno‐arterial extracorporeal membrane oxygenation.

### Mortality in relation to hospital cardiogenic shock volume

During a mean in‐hospital stay of 13.0 days (± 17.5), 130 076/220 223 patients (59.1%) with CS died. Annual in‐hospital mortality was high between 58.0% in 2017 and 61.1% in 2021 with a slight increase over time that was also observed across CS volume subgroups. Crude mortality rates were comparable between intermediate‐low (22 785/38 658, 58.9%) and high volume CS centres (107 291/181 567, 59.1%). However, after adjustment for relevant confounders, treatment at high volume CS centres was associated with a significantly lower in‐hospital mortality risk compared to intermediate‐low volume centres, with a HR of 0.92 (95% CI 0.91–0.94; *p* < 0.001).

**Figure 2:**
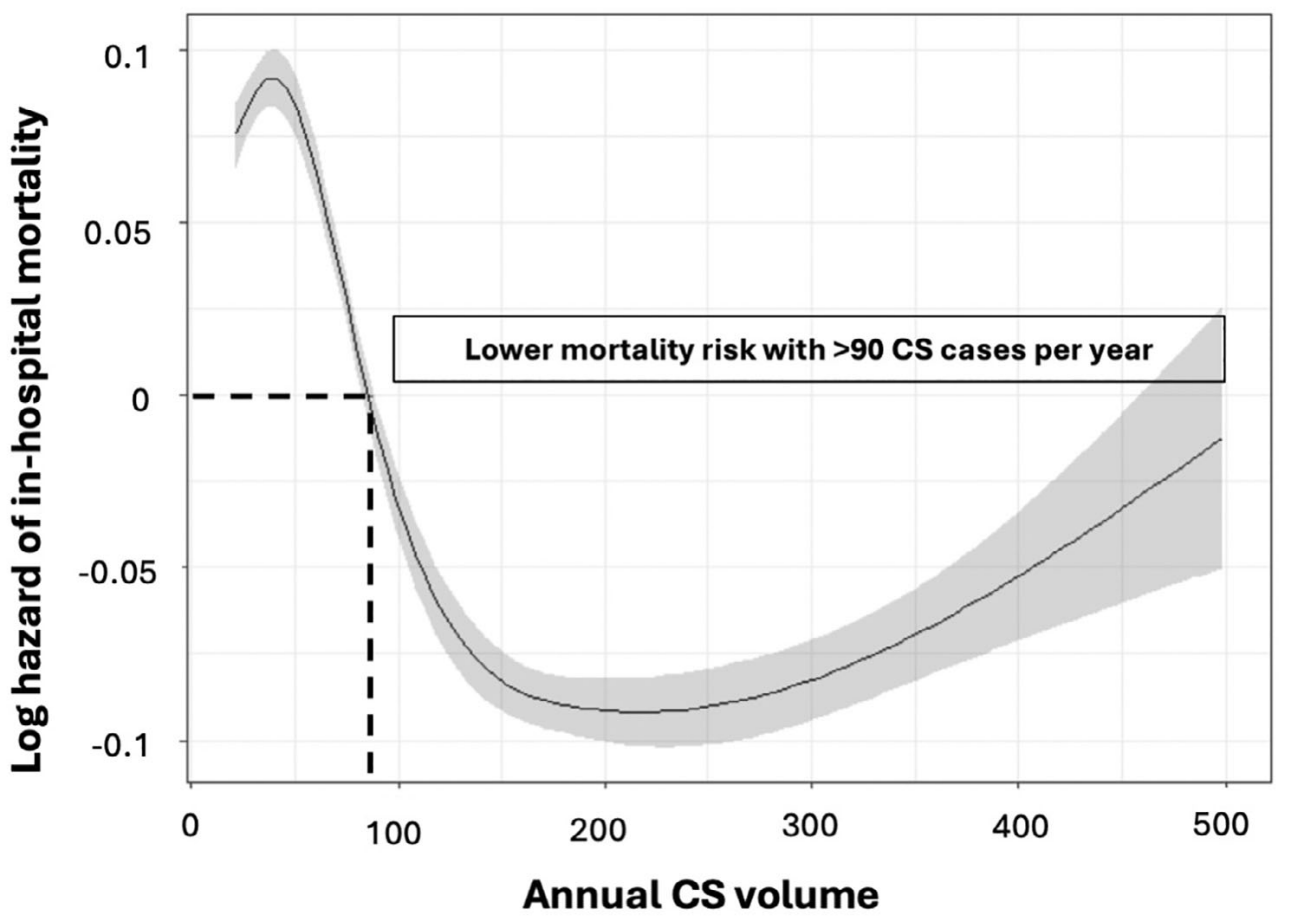
Association between annual cardiogenic shock (CS) volume and in‐hospital mortality. Cox regression model adjusted by age, sex, acute myocardial infarction, cardiopulmonary resuscitation and use of mechanical circulatory support.

**Figure 3:**
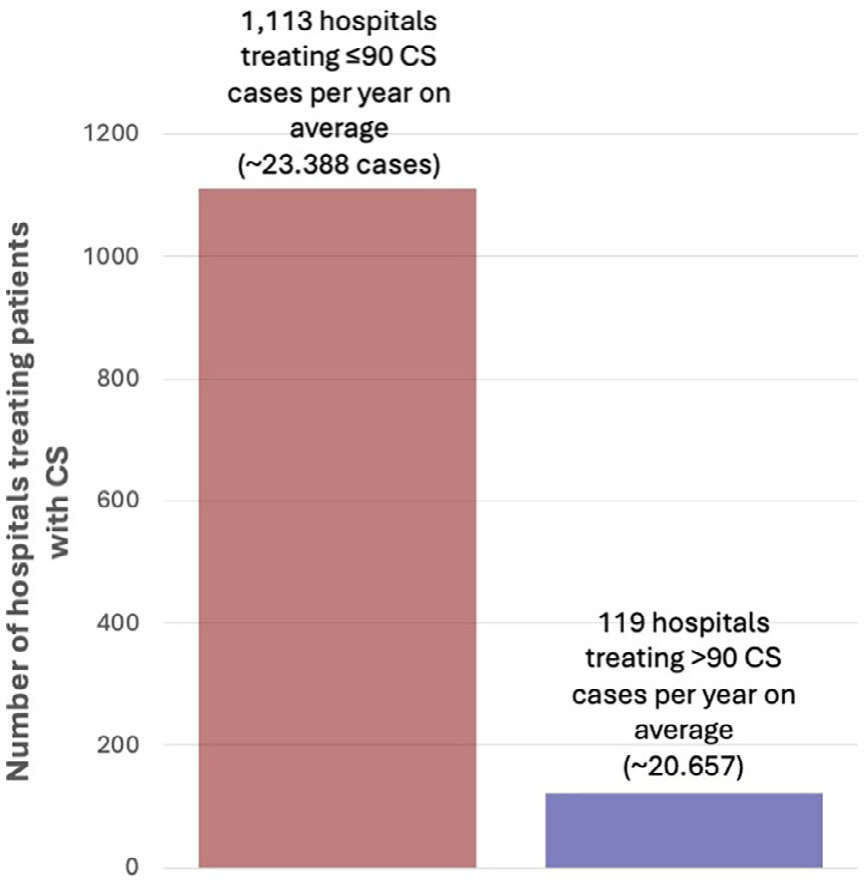
Application of the cardiogenic shock (CS) case threshold using the 5‐year mean annual CS volume in Germany.

For CS volume and mortality, cubic splines indicated a parabolic association with a steep decrease in mortality and overall lower mortality risk at >90 CS cases per year followed by an increase in mortality risk with increasing CS volume at >300 CS cases per year, which however remained associated with a lower mortality risk than a CS volume of ≤90 cases per year, as illustrated in *Figure* *2* (adjusted) and online supplementary *Figure Appendix* *S1* (unadjusted). Applying the threshold of >90 CS cases per year would exclude 1113 hospitals (90%) from CS care and redirect 23 388 CS cases (53%) on average per year to the remaining high volume hospitals (*Figure* *3*).

### Mortality in relation to hospital mechanical circulatory support volume

When treated with MCS, crude mortality was higher compared to patients not treated with MCS (62.9% vs. 58.5%). Annual in‐hospital mortality showed an increasing trend for both patient groups (60.3% in 2017 to 65.7% in 2021 for patients treated with MCS vs. 57.7% in 2017 to 60.4% in 2021 for patients treated without MCS). Crude mortality rates were comparable between intermediate‐low MCS volume centres (2131/3500, 60.9%) and high MCS volume centres (15 390/24 336, 63.2%). However, after adjustment for relevant confounders, treatment at high volume MCS centres was associated with a significantly lower in‐hospital mortality risk compared to intermediate‐low volume MCS centres, yielding a HR of 0.80 (95% C: 0.76–0.84; *p* < 0.001).

**Figure 4:**
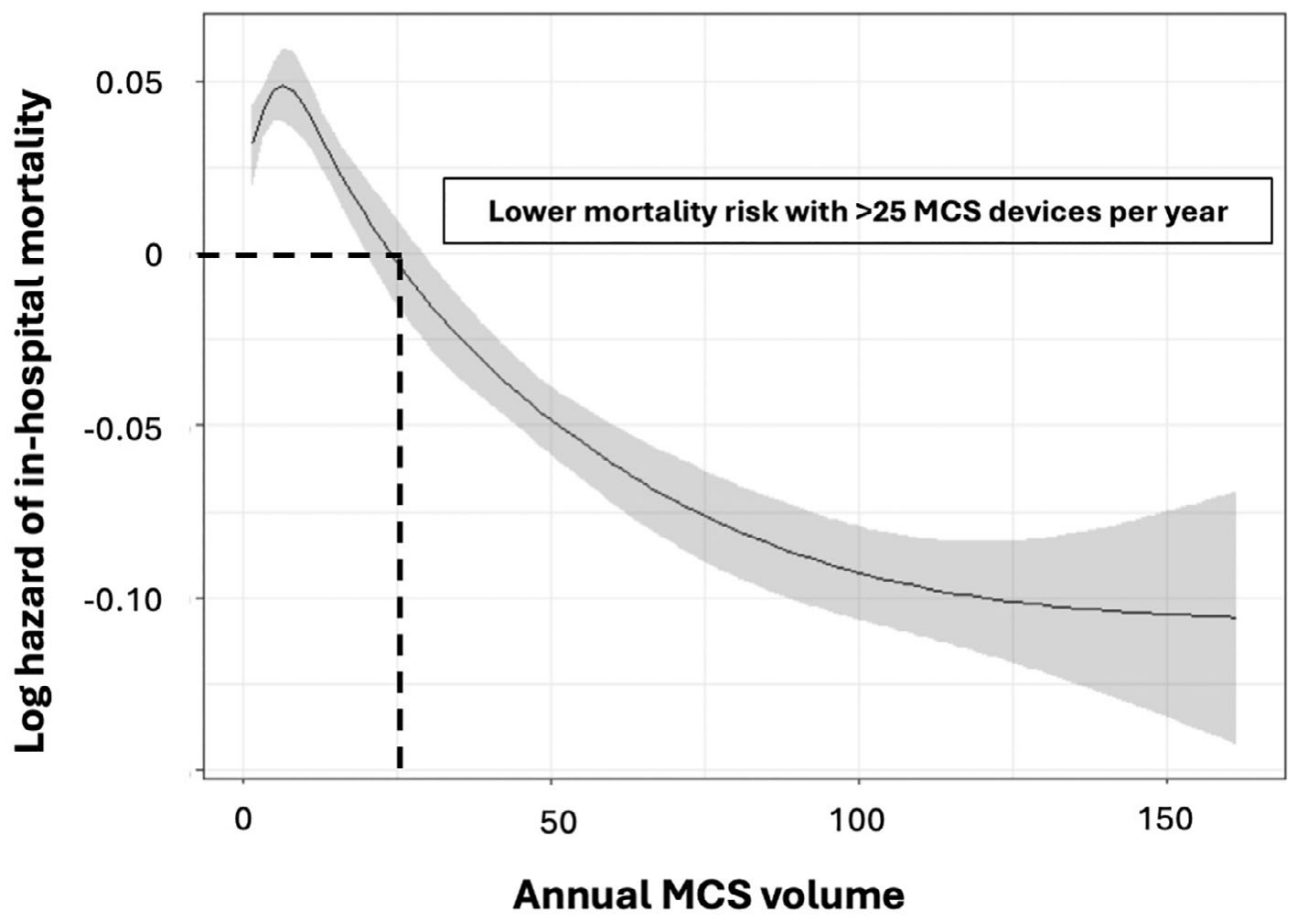
Association between annual mechanical circulatory support (MCS) volume and in‐hospital mortality. Cox regression model adjusted by age, sex, acute myocardial infarction and cardiopulmonary resuscitation.

**Figure 5:**
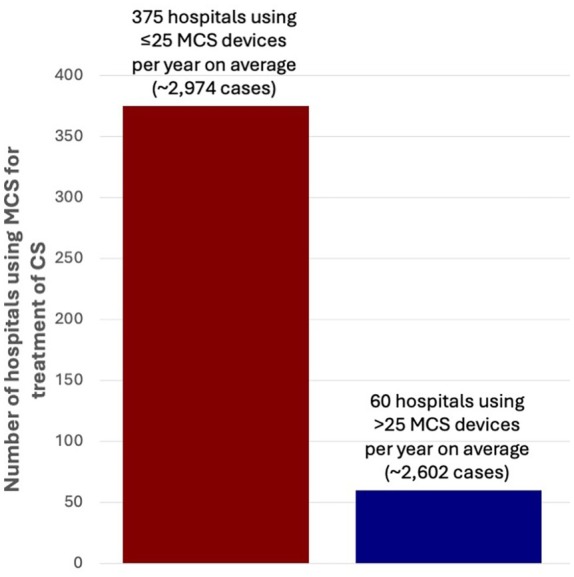
Application of the mechanical circulatory support (MCS) for treatment of cardiogenic shock (CS) case threshold using the 5‐year mean annual MCS volume in Germany.

Cubic spline plot analyses indicate a linear association between MCS volume and lower mortality risk for patients treated in hospitals with an annual MCS volume of >25 cases per year, illustrated in *Figure* *4* (adjusted) and online supplementary *Figure* *S2* (unadjusted). Applying the threshold of >25 MCS cases per year would exclude 375 hospitals (86%) from MCS use for treatment of CS and redirect 2974 MCS cases (53%) on average per year to the remaining high volume hospitals (*Figure* *5*).

## Discussion

This study underscores the latent advantages of centralizing care to enhance outcomes for CS care and use of MCS on a national scale: among 220 223 consecutive CS cases, treatment in hospitals with higher annual volume of both CS care and use of MCS was associated with a lower mortality risk. The current landscape, however, is far from centralized, particularly concerning MCS use. Alarmingly, 86% of hospitals employing MCS devices handle fewer than 25 cases annually, and 66% handle fewer than 8 cases annually, resulting in suboptimal outcomes. The data suggest that establishing minimum case thresholds—90 cases per year for CS care and 25 cases per year for MCS use—could not only foster resource‐effective centralization but ultimately enhance patient outcomes (*Graphical Abstract*).

To address the high mortality risk of CS, the use of MCS has increased in recent years, mostly as a bridge‐to‐therapy option, e.g. to buy time for other treatments, and partly as a treatment itself, e.g. to minimize myocardial damage.^1^, ^5^, ^16^ However, our analysis shows that the in‐hospital mortality rate of CS remains unacceptably high at 59% over the years, which is consistent with previous reports.^1^, ^2^, ^17^ Hence, new approaches are needed to tackle this ongoing, resource demanding and impactful healthcare problem.

Among those is the idea of rethinking CS care and MCS use on a regional level. Especially since the success of the DanGer Shock trial a focus has been laid on a rigorous patient selection and the application of standardized treatment protocols, aiming to minimize complications and treatment heterogeneity.^6^ Interestingly, applying the same rigorous selection criteria from the DanGer Shock trial to the ECLS‐Shock population seems to identify patients who are likely to benefit from VA‐ECMO.^7^ These findings are an indicator that improvement in CS care should not only be sought by identifying new treatment strategies, but especially by improving the application of existing strategies. This is in line with societies calling for an increasing emphasis on multidisciplinary teams and the establishment of dedicated CS networks, centralizing treatment in few dedicated, highly experienced and ultimately more effective centres.^3^, ^18^, ^19^, ^20^, ^21^

In contrast to this, we observed a wide variation in the distribution of CS and MCS cases across Germany. Currently, most hospitals manage only few CS cases and have an extremely low MCS use per year, while only a minority manages high volumes. Even more concerning is the current trend that the high volume hospitals seem to become more selective with the use of MCS devices, whereas intermediate‐low volume hospitals seem to become more liberal. This is likely supported by the relatively low structural barriers to implement MCS use in Germany, as in principle all hospitals which offer primary percutaneous coronary intervention are also able to use one of the three main MCS devices, with complete coverage by the health insurance system. Also, there are no other ubiquitous contraindication in relation to MCS use in Germany, e.g. no strict upper age limit, which may also contribute towards a more liberal use of MCS in Germany.

This trend toward a decentralization of MCS care stands in stark contrast to the clear volume–outcome association shown in this study: not only was treatment in a high volume CS centre associated with a 8% lower relative mortality risk, but even more astonishing is the 20% relative mortality risk reduction seen when MCS devices were used in high volume centres without signs of a ceiling effect. The strength of this association comes close to the one observed in the DanGer Shock trial, but on a national level and thereby at a far larger scale.^6^ Although previous studies have investigated the volume–outcome association in CS sub‐groups or for general CS management, none has shown this in a contemporary, nationwide cohort or for MCS use.^22^, ^23^ Several factors might help to explain this reduced mortality risk in high volume centres. Among those, more effective treatment of the underlying disease could be one factor (e.g. higher likelihood to use percutaneous coronary intervention and coronary artery bypass graft as seen in this study). However, it is more likely that higher hospital experience translates into improved, standardized and better implemented processes around CS care and especially MCS use. In the end, MCS implantation only requires few experienced operators, but their management a large and consistently trained team.

These findings indicate a potential benefit of a centralization of CS care and MCS use, creating few specialized centres with subsequently high expertise, experience, and performance standards. By strategically distributing resources on a national level, we have the chance to fully exploit the linear association between high volumes and low mortality risk. Our analysis indicates that the minimum case threshold for CS care should be at least 90 cases per year, and for MCS use at least 25 cases per year. Applying these thresholds would significantly reshape the CS and MCS landscape in Germany, limiting the number of hospitals to 10% of its current value for CS care and to 16% for MCS use. As a result, approximately 50% of CS and MCS cases would be redirected to the remaining high volume centres. However, it needs to be kept in mind that also the resources of sufficiently funded high volume hospitals are limited, so that ultimately the redirection of CS patients in need or likely need of MCS should be prioritized over the redirection of all CS patients, e.g. also those not in need of MCS or not eligible for MCS use. This would allow to strategically relocate resources, is likely to reduce costs, and could potentially contribute to improve patient outcomes.

An important argument that is made when discussing regional organization of care and volume–outcome relationships is that of accessibility and feasibility. Treatment should be delivered according to patients needs in the most suitable centre but also in a timely manner.^15^ However, several studies have shown no association between patient‐hospital distance and outcomes in this setting which justifies centralization strategies for CS and MCS patients.^23^, ^24^, ^25^

Overall, to improve CS care and MCS use, we advocate for the centralization of CS care and especially MCS use through a hub‐and‐spoke network structure. This approach aims to ensure that patients receive care from facilities equipped with the necessary expertise, experience and resources to effectively manage these high‐risk cases.

### Limitations

The primary strength of this study lies in the analysis of a complete, nationwide, contemporary data set. This provides reliable mortality information, temporal trends, an ability to correct for known factors influencing outcome. However, this study is subject to several limitations. First of all, it is important to highlight that this study evaluated the association between hospital volumes and outcomes, but not of an actual centralization of care, which limits the direct applicability of our findings to this topic. As an observational study with non‐randomized data there is risk for unmeasured and unknown confounders as well as selection bias. Furthermore, data stem from the German governmental coding system, with case definitions dependent on ICD‐10‐GM and OPS codes, and potential for oversimplification. While reimbursement‐relevant characteristics such as the use of MCS are reported with high accuracy, non‐reimbursement‐relevant characteristics likely accompanying baseline characteristics are underreported. Information on outcome modulators in CS, including lactate, shock severity, and other features, were not available. Another consideration is that by design of the underlying data source, cases cannot be linked across different hospital stays or followed up after discharge for data privacy reasons, so that the same patient may have multiple entries in the database from different hospitals, without the possibility to provide detailed data on intra‐hospital transfers or long‐term outcomes. Importantly, we lack information regarding the referral of patients between hospitals, which might have diluted the volume–outcome association: some patients might have been initially treated and implanted with an MCS device in an intermediate‐low volume hospital, but were then transferred to a high volume hospital. If those patients died in the high volume hospital, they would show as ‘survivors’ for the intermediate‐low volume hospital and as ‘non‐survivors’ for the high volume hospital. This effect could partially explain the unexpected rise in mortality with CS (but not with MCS) in the centres with the highest volume of patients. Lastly, although the data are particularly valid within the German context, generalization to different healthcare systems might be limited.

## Conclusion

The current landscape of CS care and MCS use in Germany is fragmented, as most hospitals treat only few cases per year, which was associated with inferior outcomes as compared to treatment in high volume centres. These findings indicate that centralization of CS care in specialized centres treating high volumes of patients with CS and MCS might improve outcomes. All relevant stakeholders, ranging from healthcare policymakers to physicians and patients, need to engage in a discussion on this topic, ultimately aiming to restructure and improve CS care on a national scale.

## Supporting information


**Appendix S1.**Supporting Information.
