# Supplementary material for: Higher hospital volume is associated with lower mortality for patients with cardiogenic shock and mechanical circulatory support
Source: Eur J Heart Fail. 2025 Aug 31;27(11):2074–83. doi: 10.1002/ejhf.70025 (PMC12766554; doi:10.1002/ejhf.70025)
Supplement: Supplementary file 1 — Appendix S1.Supporting Information. [file EJHF-27-2074-s001.docx]

***SUPPLEMENTARY APPENDIX***

***Supplementary Table 1.*** Variable Definitions based on ICD-10-GM and OPS codes.

| **Variable** | **Definition** |
| --- | --- |
| Cardiogenic Shock | ICD-10-GM: R57.0 |
| Atrial fibrillation | ICD-10-GM: I48 |
| Diabetes mellitus | ICD-10-GM: E10, E11 |
| Arterial hypertension | ICD-10-GM: I10 |
| Dyslipidemia | ICD-10-GM: E78 |
| Chronic heart failure | ICD-10-GM: I50 |
| Coronary artery disease | ICD-10-GM: I25 |
| History of CABG | ICD-10-GM: Z95.1 |
| Peripheral artery disease | ICD-10-GM: I70.2 |
| Prior stroke | ICD-10-GM: I63, I64 |
| Pulmonary hypertension | I27.0, I27.2, I27.20 |
| COPD | ICD-10-GM: J44 |
| Chronic kidney disease | ICD-10-GM: N18, N19 |
| Acute myocardial infarction | ICD-10-GM: ICD I21 |
| Severe pulmonary embolism | ICD-10-GM: I26.0 |
| Acute myocarditis | ICD-10-GM: I40, I41 |
| Peripartum cardiomyopathy | ICD-10-GM: O99.4, O90.3 |
| Post cardiothoracic surgery | ICD-10-GM: I97 |
| Cardiac arrest | ICD-10-GM: U69.13, OPS 8-77 |
| Coronary angiogram | OPS: 1-275 |
| Percutaneous coronary intervention | OPS: 8-837 |
| CABG | OPS: 5-361, 5-362 |
| Mechanical circulatory support | OPS: 8-839.0, 8-839.4, 8-839.a, 8-852.3 |
| IABP | OPS: 8-839.0 |
| mAFP | OPS: 8-839.4 |
| VA-ECMO | OPS: 8-839.a, 8-852.3 |
| Renal replacement therapy | OPS: 8-853, 8-854, 8-855 |
| Invasive ventilation | OPS: 8-701 |

ICD-10-GM, German modification of the International Statistical Classification of Diseases and Related Health Problems, 10th revision; OPS, German Operational and Procedural codes; CABG, coronary artery bypass graft; COPD, chronic obstructive pulmonary disease; CS, cardiogenic shock; IABP, intra-aortic balloon pump; mAFP, micro-axial flow pump; MCS, mechanical circulatory support; VA-ECMO, veno-arterial extracorporeal membrane oxygenation.

***Supplementary Table 2.*** Yearly MCS volume per device from 2017 to 2021

|  | **No MCS**  **(N=192,387)** | **MCS**  **(N=27,836)** | **IABP**  **(N=4,607)** | **mAFP**  **(N=12,454)** | **VA-ECMO**  **(N=10,775)** |
| --- | --- | --- | --- | --- | --- |
| **2017** | 40,389 (88.8) | 5,085 (11.2) | 1,389 (3.1) | 1,707 (3.8) | 1,989 (4.4) |
| **2018** | 38,707 (87.7) | 5,439 (12.3) | 1,023 (2.3) | 2,322 (5.3) | 2,094 (4.7) |
| **2019** | 38,863 (86.9) | 5,845 (13.1) | 885 (2.0) | 2,624 (5.9) | 2,336 (5.2) |
| **2020** | 37,453 (86.9) | 5,671 (13.5) | 704 (1.6) | 2,732 (6.3) | 2,235 (5.2) |
| **2021** | 36,975 (86.5) | 5,796 (13.6) | 606 (1.4) | 3,069 (7.2) | 2,121 (5.0) |

Values are shown as frequency (with percentage). Variables were compared using χ2 test. IABP, intra-aortic balloon pump; mAFP, micro-axial flow pump; MCS, mechanical circulatory support; VA-ECMO, veno-arterial extracorporeal membrane oxygenation.

***Supplementary Figure 1.***

**
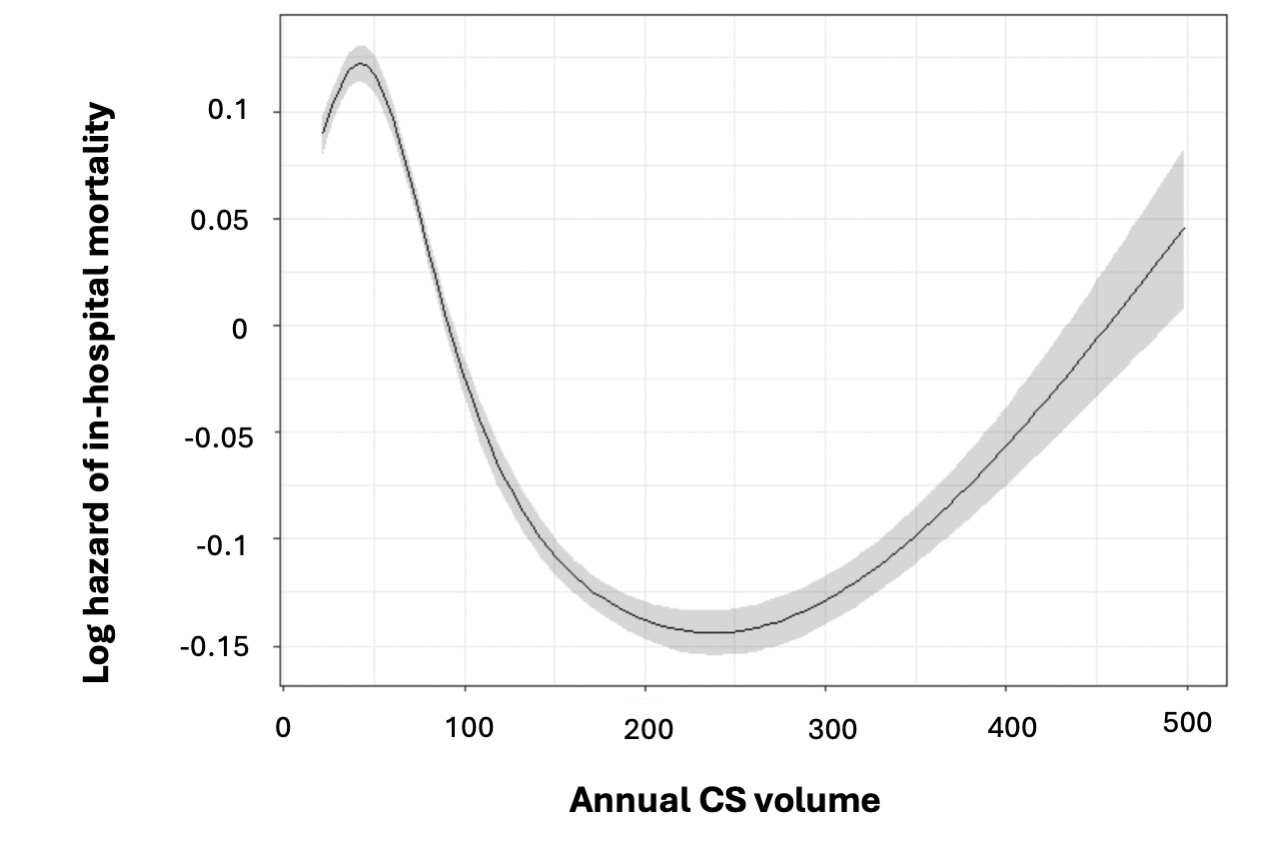
**

Association between annual cardiogenic shock (CS) volume and in-hospital mortality. Unadjusted Cox regression model.

***Supplementary Figure 2.***

**
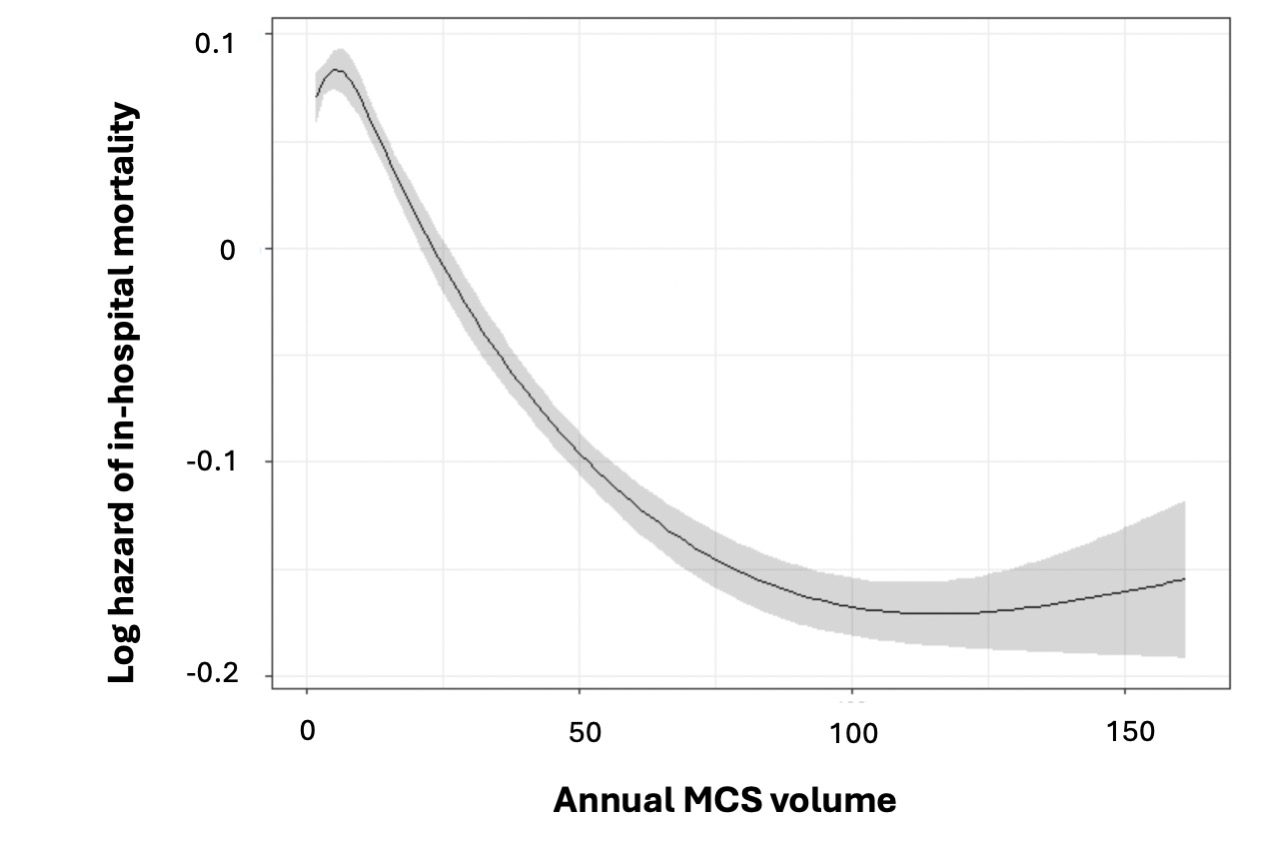
**

Association between annual mechanical circulatory support (MCS) volume and in-hospital mortality. Unadjusted Cox regression model.
